# Anti-Inflammatory Neutrophils Reprogram Macrophages toward a Pro-Healing Phenotype with Increased Efferocytosis Capacity

**DOI:** 10.3390/cells13030208

**Published:** 2024-01-23

**Authors:** Andreea Cristina Mihaila, Letitia Ciortan, Monica Madalina Tucureanu, Maya Simionescu, Elena Butoi

**Affiliations:** Biopathology and Therapy of Inflammation, Institute of Cellular Biology and Pathology “Nicolae Simionescu”, 050568 Bucharest, Romania; andreea.mihaila@icbp.ro (A.C.M.); letitia.ciortan@icbp.ro (L.C.); maya.simionescu@icbp.ro (M.S.)

**Keywords:** neutrophil secretome, pro-healing macrophages, efferocytosis, MerTK, bridge molecules

## Abstract

Following myocardial infarction (MI), blood neutrophils quickly and extensively infiltrate the heart, where they are temporally polarized into pro-inflammatory (N1) and anti-inflammatory (N2) subpopulations. Neutrophil transmigration is rapidly followed by the accrual of macrophages (MACs), which are believed to undergo local phenotypic transformations from pro-inflammatory to pro-healing MACs that mediate inflammation resolution. We hypothesized that N2 neutrophils can reprogram MACs toward a healing phenotype with increased efferocytosis capacity. To examine this, human neutrophils isolated from healthy subjects were polarized in N1 and N2 neutrophils, and their secretome was added to human MACs derived from THP monocytes. The impact of neutrophil factors on macrophages was investigated using qPCR, ELISA, Western blot, immunofluorescence, or an efferocytosis assay. The results show that the MACs exposed to N2 neutrophil secretome exhibited (i) increased expression of the anti-inflammatory molecules *CD206*, TGF-β, and IL-10 and the nuclear factors associated with reparatory macrophages (PPARγ, Nur77, and *KLF4*); (ii) enhanced expression of efferocytosis receptors (MerTK, *CD36*, CX3CR1, and *integrins αv/β5*) and of the bridge molecules Mfage8 and Gas6; and (iii) enhanced efferocytosis. In conclusion, factors released by N2 neutrophils induce a pro-healing phenotype of MACs by upregulating anti-inflammatory molecules and efferocytosis receptors and ensuing the efferocytosis capacity. The data suggest that molecular therapy to foster N2 polarization, which boosts macrophages’ pro-healing phenotype, could be a promising strategy to speed up inflammation resolution and tissue repair.

## 1. Introduction

Macrophages (MACs) are tissue-resident or -infiltrating immune cells with a determinant role in normal tissue development, innate immunity, and repair of injured tissue. They can detect and respond to various pathogens and environmental stimuli, thus playing a key role in the body’s immune response. Furthermore, they play a crucial part in tissue repair processes following injury, including cardiac damage [1].

Myocardial infarction (MI) is the most common cause of cardiac injury, and subsequent reperfusion further enhances the activation of innate and adaptive immune responses and cell death programs. Within the first 24 h of ischemia’s onset, the first immune cells that reach the injured area are neutrophils, which are attracted by cell debris and inflammatory molecules released by apoptotic cardiomyocytes. They massively infiltrate the infarct area to coordinate the initial pro-inflammatory response. They secrete granule components, such as myeloperoxidase and proteases, and produce high levels of reactive oxygen species (ROS), thereby exacerbating local tissue injury [2]. Subsequently, neutrophils pave the way for the infiltration of blood monocytes, which become macrophages and help to remove cardiac tissue debris and apoptotic cells, leading, in turn, to activation of the reparative pathways required for cardiac remodeling [3]. While innate immune cells play a crucial role in cardiac healing, an imbalanced or exaggerated immune response following MI, as well as the defective plasticity of these cells, can exacerbate tissue damage and, subsequently, trigger maladaptive remodeling [4,5].

In the ischemic myocardium, MACs demonstrate a remarkable degree of plasticity and are intricately involved in both inflammatory and reparative processes [6]. Macrophages are commonly classified into two distinct groups, classically activated—M1 (inflammatory) MACs and alternatively activated—M2 (anti-inflammatory) MACs, although the phenotypic profile of cardiac MACs is much more diverse. While M1 MACs are mainly involved in phagocytosis and exacerbation of inflammation [7], M2 macrophages stimulate tissue repair and regeneration and have efferocytic, pro-fibrotic, and pro-angiogenic properties [8]. Therefore, the course of the polarization of macrophages post-MI plays a crucial role in inflammation resolution, tissue regeneration, and the wound healing process [9].

Post-MI, during the process of inflammation resolution, pro-inflammatory MACs are believed to undergo a local transformation into MACs that mediate resolution. Recruited and resident MACs are continuously exposed to both damage-associated molecular patterns (DAMPs) released by apoptotic cardiomyocytes and to various granular components and pro-inflammatory molecules produced by neutrophils. The granule content of neutrophils not only contributes to ischemic damage but also facilitates the recruitment of monocytes, and promotes the reprogramming of MACs toward a resolving phenotype [10]. However, the direct effect of the factors released by neutrophil subtypes on the MAC phenotype and functionality is not well known.

Previous data showed that in the first days post-MI, the dominant neutrophils found in the infarcted left ventricle are of the N1 pro-inflammatory subtype [11]. The transcriptional and functional analysis we recently performed showed that N1 neutrophils exhibit: increased production of inflammatory cytokines, elevated levels of ROS and nitric oxide, augmented oxidative burst, increased activity of protein- and matrix-degrading enzymes, as well as enhanced chemotactic response [12]. Starting on day 5, the N2 anti-inflammatory neutrophil subtype was identified in the infarcted area, and their number was picked on day 7 [11]. Regarding this subtype, our previous work showed that N2 neutrophils display increased expression of the anti-inflammatory markers *CD206*, *Ym1*, and *Arg1*, and have similar ROS, nitric oxide, and chemotactic responses as the un-activated, control neutrophils [11]. Because, following MI, the macrophages are exposed initially to factors released by inflammatory N1 neutrophils, and after 5–7 days the MACs also sense the factors released by N2 neutrophils, we questioned the impact of N1/N2 neutrophils on efferocytosis—a key function of macrophages in the cardiac lesions. In different cardiac injury models, previous studies have shown that the depletion of MACs has a deleterious effect, resulting in the inability to clear tissue debris and dead cells, including necrotic cardiomyocytes [1,13]. The MACs capacity to clear apoptotic cells and debris—the efferocytosis process—is mainly regulated by: (i) soluble factors that attract macrophages to the site of cell death (find-me molecules), (ii) efferocytosis receptors that facilitate recognition of dead cells by phagocytes (bridging molecules and receptors), and (iii) intracellular molecular pathways that regulate the engulfment [14]. The targeting of genes that support efferocytosis, such as Mer proto-oncogene tyrosine kinase (*MerTK*) and milk fat globule-EGF factor 8 (*MFG-E8*), results in impaired tissue healing and heart failure after cardiac injury, indicating the essential role of clearing tissue debris in healing following cardiac injury [1,13].

Here, we provide evidence that mediators released by neutrophil subtypes have a significant effect on the phenotypic modulation of macrophages. The factors released by N1 neutrophils induce the transition of MACs toward the M1 phenotype, whereas when exposed to N2 secretome, MACs turn into a pro-healing phenotype, manifested by increased expression of anti-inflammatory markers and enhanced receptor-mediated efferocytosis.

## 2. Materials and Methods

### 2.1. Isolation and Polarization of Neutrophils

Human neutrophils were isolated from the peripheral blood of healthy volunteers within 60 min after blood collection, using the MACSxpress^®^ Whole Blood Neutrophil Isolation Kit (Miltenyi, Bergisch Gladbach, Germany), or Polymorphprep (ProteoGenix, Schiltigheim, France) as per the manufacturer’s instructions. Subsequently, the neutrophils were washed with HBSS and resuspended in RPMI 1640 medium (10 × 10^6^ neutrophils/mL). The cells were polarized into the N1/N2 subtypes, by exposure for 2 h to 100 ng/mL lipopolysaccharide (LPS) and 20 ng/mL interferon gamma (IFNγ) or 20 ng/mL interleukin 4 (IL-4), respectively, as we previously described [12]. After 2 h, neutrophils were centrifuged and the fresh medium was added for another 18 h. At the end of the incubation time, the conditioned media (secretome) containing factors released by N1 or N2 neutrophils was collected and used for further experiments. To verify if the polarization protocol used previously for mouse neutrophils induces the N1/N2 phenotype in human neutrophils, the main inflammatory/anti-inflammatory markers for each phenotype were analyzed. The qPCR results show that, like mouse neutrophils, LPS + INFγ induced an N1—inflammatory phenotype and IL-4 an anti-inflammatory phenotype to human neutrophils (Supplementary Materials Figure S1).

The study was approved by the Institutional Ethics Committee of ICBP “N. Simionescu” (Bucharest, Romania), and the investigation was carried out according to the principles outlined in the Declaration of Helsinki [15].

### 2.2. Macrophage

THP1 human monocytic cells (from ATCC) derived from an acute monocytic leukemia patient were cultivated at 37 °C in RPMI-1640 medium containing 10% heat-inactivated FBS, 100 U/mL penicillin, and 100 μg/mL streptomycin in a humidified incubator with 5% CO_2_. THP1 cells were differentiated into macrophages by incubation with 100 nM phorbol 12-myristate 13-acetate (PMA) for 72 h before the experiments.

### 2.3. Experimental Design

To investigate the impact of the factors secreted by N1/N2 neutrophils on macrophages derived from THP1 monocytes, the secretome was collected from N1/N2 neutrophils (obtained as described in Section 2.1) and added to the macrophages for 24/72 h. After specific incubation time, the conditioned media and the macrophages were isolated and prepared for further analysis. In some experiments, the secretome of N2 neutrophils was added onto macrophages in the presence of GW9662 (10 µM), a peroxisome proliferator-activated receptor gamma (PPARγ) specific antagonist.

### 2.4. RNA Extraction and Quantitative RT-PCR

Total RNA was isolated from macrophages using Qiagen PureLink RNA Kit (Ambion™, Carlsbad, CA, USA) or TRIzol. Approximately 1 µg of total RNA was used for the cDNA synthesis with MMLV reverse transcriptase, according to the manufacturer’s protocol (Invitrogen, Waltham, MA, USA). mRNA expression was assessed by amplification of cDNA using the LightCycler 480 Real-Time PCR System (Roche, Basel, Switzerland), with specific primers and SYBR Green I. The sequences of used primers are shown in Supplementary Materials Table S1. The relative quantification was performed using the comparative CT method and expressed as arbitrary units. Beta 2 microglobulin was used as the reference gene.

### 2.5. Enzyme-Linked Immunosorbent Assay (ELISA)

The supernatant was isolated from polarized N1 and N2 neutrophils or macrophages exposed to the neutrophil secretome. The amounts of the proteins of interest—S100A8/A9, tumor necrosis factor (TNF-α), interleukin-10 (IL-10), transforming growth factor-beta (TGF-β1), and growth-arrest-specific protein 6 (Gas6)—released by macrophages or neutrophils were measured using specific kits (R&D Systems, Minneapolis, MN, USA), following the manufacturer’s instructions.

### 2.6. Western Blot Analysis

Following exposure to conditioned media from polarized neutrophils, macrophages were harvested in RIPA lysis buffer containing a protease inhibitor cocktail. After sonication and centrifugation (12,000× *g*), the quantity of proteins was quantified with a bicinchoninic acid (BCA) assay kit. Proteins from each sample (30 mg) were separated upon 10% SDS-PAGE (sodium dodecyl sulfate-polyacrylamide) gel electrophoresis, followed by transfer to nitrocellulose membranes. The resulting blots were, subsequently, probed with specific primary antibodies directed against IL-1β (MAB601, R&D Systems, Minneapolis, MN, USA), caspase 1 (MAB6215, R&D Systems, Minneapolis, MN, USA), TGF-β1 (MAB240, R&D Systems, Minneapolis, MN, USA), MerTK (WH0010461M1, Sigma-Aldrich, Taufkirchen, Germany), CX3CR1 (AF5825, R&D Systems, Minneapolis, MN, USA), MFG-E8 (PA5-109955, Invitrogen, Waltham, MA, USA), pERK1/2 (#4377S, Cell Signaling, Danvers, MA, USA), p38MAPK (AF869, R&D Systems, Minneapolis, MN, USA), pp38MAPK (#4511, Cell Signaling, Danvers, MA, United States), PPARγ (sc-7196, Santa Cruz Biotechnology, Heidelberg, Germany), CX3CR1 (sc-30030, Santa Cruz Biotechnology, Heidelberg, Germany), and β-actin (A3854, Sigma-Aldrich, Taufkirchen, Germany), followed by the secondary antibodies including anti-rabbit HRP-coupled (HAF008, R&D Systems, Minneapolis, MN, USA), anti-mouse HRP-coupled (#31430, R&D Systems, Minneapolis, MN, USA), or anti-goat HRP-coupled (HAF017, R&D Systems, Minneapolis, MN, USA). A SuperSignal West Pico chemiluminescent substrate (Pierce, Rockford, IL, USA) was used to visualize the signals and a Luminescent image analyzer, the LAS 4000 (Fujifilm, Freiburg, Germany), with the Image reader LAS 4000 software to quantify the signals.

### 2.7. Flow Cytometry

Apoptosis of cardiomyocytes was assessed with the FITC Annexin V Apoptosis Detection Kit with PI (BioLegend, San Diego, CA, USA). Upon staurosporine treatment, cardiomyocytes were washed with cold cell staining buffer and resuspended in Annexin V Binding Buffer with 5 µg/mL Annexin V-FITC and 10 µg/mL of PI. Samples were incubated at room temperature in the dark for 15 min followed by flow cytometry analysis using the Cytoflex flow cytometer (Beckman Coulter, Brea, CA, USA).

### 2.8. MerTK Immunofluorescence

After incubation with secretome from N1/N2 neutrophils, macrophages were washed twice with HBSS and fixed with 4% paraformaldehyde and blocked with 5% powdered milk, 1% fish gelatin, 1% BSA, and 0.2% Tween 20. Cells were incubated overnight with primary anti-human MerTK antibody (WH0010461M1, Sigma), followed by incubation with rabbit anti-mouse IgG-FITC secondary antibody (sc-358916, Santa Cruz). Nuclei were stained with DAPI, and images were acquired using a fluorescence microscope, the Olympus IX8 equipped with an XM10 camera, and processed using ImageJ 1.54f software.

### 2.9. In Vitro Efferocytosis Assay

To induce the apoptosis of HL-1 cardiomyocytes, cells were seeded in 6-well plates in Claycomb medium and exposed to 1 and 3 µM staurosporine for 5 h at 37 °C with 5% CO_2_. Cardiomyocyte apoptosis was confirmed by Annexin V staining using flow cytometry. Human macrophages derived from THP1 monocytes, previously exposed to the N1/N2 secretome, were incubated with PKH-labeled apoptotic cardiomyocytes (PKH26GL-1KT, Sigma) at a 1:3 ratio for 3 h. Upon the removal of the non-phagocytosed cells, the macrophages were stained with anti-human MerTK (as described below) and analyzed by fluorescence imaging using a fluorescence microscope, the Olympus IX81 (Olympus, Tokyo, Japan) equipped with an XM10 camera (Olympus, Tokyo, Japan), and processed using ImageJ 1.54f software.

### 2.10. Statistical Analysis

All statistical analyses were performed with GraphPad Prism 7.0 software with the data points denoting the mean ± standard deviation (SD). Statistical significance is shown as *p*-values obtained via a two-tailed Student’s *t*-test when comparing two experimental groups and analysis of variance (one-way ANOVA–Bonferroni) with multiple comparisons when comparing more than two groups. The *n* from figure legends indicates three separate experiments. Asterisks indicate significant differences among experimental groups (* *p* < 0.05, ** *p* < 0.01, *** *p* < 0.001).

## 3. Results

### 3.1. Factors Released by N1/N2 Neutrophils Differently Modulate the Macrophage Expression of Inflammatory/Anti-Inflammatory Markers 

**Figure 1:**
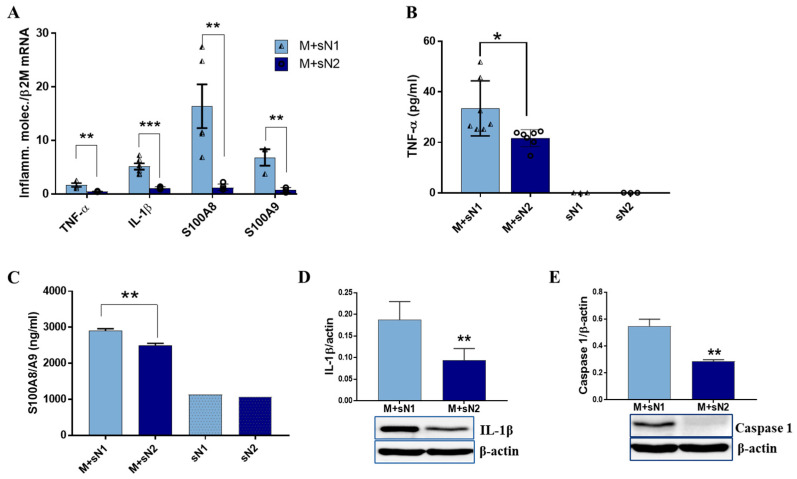
Inflammatory markers were induced in macrophages exposed to factors released by N1 neutrophils. (**A**) Comparative gene expressions of *TNF-α*, *IL-1β*, *S100A8*, and *S100A9* in macrophages exposed to the secretome collected from N1 (M + sN1) or N2 neutrophils (M + sN2). (**B**,**C**) Comparative profile of TNF-α and S100A8/A released in conditioned media of macrophages (M + sN1 and M + sN2) and in N1/N2 neutrophil secretome, by ELISA. The data represent the mean ± SD from four different experiments. (**D**,**E**) Protein expression of IL-1β and caspase 1 in the lysate of M + sN1 and M + sN2, by Western blot. The data represent the mean ± SD of three different experiments ** *p* < 0.01, *** *p* < 0.001 (M + sN1 versus M + sN2).

**Figure 2:**
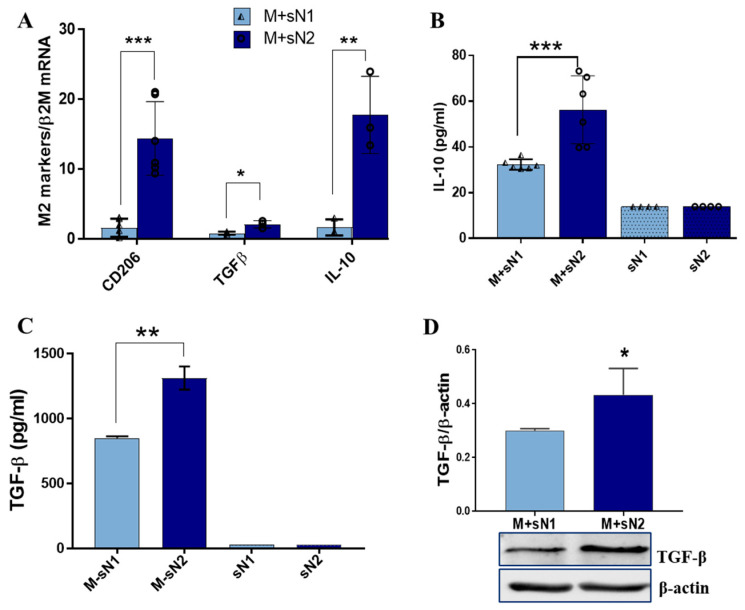
Anti-inflammatory markers were induced in macrophages exposed to factors released by N2 neutrophils. (**A**) Gene expression of *CD206*, *TGF-β*, and *IL-10* in macrophages exposed to secretome of N1 (M + sN1) or N2 neutrophils (M + sN2). (**B**,**C**) Measurement of IL-10 and TGF-β released in the conditioned media of macrophages (M + sN1 and M + sN2) and in N1/N2 neutrophil secretome, by ELISA. The data represent the mean ± SD of four different experiments. (**D**) Protein expression of TGF-β in the lysate of M + sN1 and M + sN2 macrophages. The data represent the mean ± SD of three different experiments, * *p* < 0.05, ** *p* < 0.01, *** *p* < 0.001 (M + sN1 versus M + sN2).

To assess the effect of factors released by N1/N2 neutrophils on the macrophage phenotype, the expression of some pro-inflammatory/anti-inflammatory markers was quantified. The qPCR analysis revealed that MACs derived from THP1 monocytes exposed to secretome derived from N1 neutrophils (sN1) exhibited higher gene expression of the pro-inflammatory mediators *TNF-α*, *IL-1β*, *S100A8*, and *S100A9* (Figure 1A) than MACs incubated with secretome amassed from N2 neutrophils (sN2). The protein assays (ELISA) corroborated well with the gene expression data, as soluble TNFα and S100A8/A9 were released into the conditioned media at significantly higher levels by the MACs exposed to sN1 versus sN2 (Figure 1B,C). In addition, IL-1β protein expression in cell lysate significantly increased in cells exposed to sN1 (Figure 1D) and was accompanied by an increased protein expression of caspase 1 (Figure 1E)—an inflammatory mediator that cleaves and activates IL-1β [16]. These results suggest that factors released by N1 neutrophils polarize macrophages toward a pro-inflammatory phenotype. In contrast, investigation of the anti-inflammatory markers revealed that factors released from N2 neutrophils induce an anti-inflammatory response in macrophages. Thus, the gene expressions of *CD206*, *TGF-β*, and *IL-10* were significantly upregulated in M + sN2 compared with M + sN1 (Figure 2A). In good agreement with these data, the levels of IL-10 and TGF-β protein released in the conditioned media by M + sN2 were significantly higher (Figure 2B,C), and the protein expression of TGF-β in the cell lysate significantly increased upon incubation with sN2 (Figure 2D).

### 3.2. Macrophages Exposed to Factors Released by N2 Neutrophils Upregulate Receptors Involved in the Recognition of Apoptotic Cells

**Figure 3:**
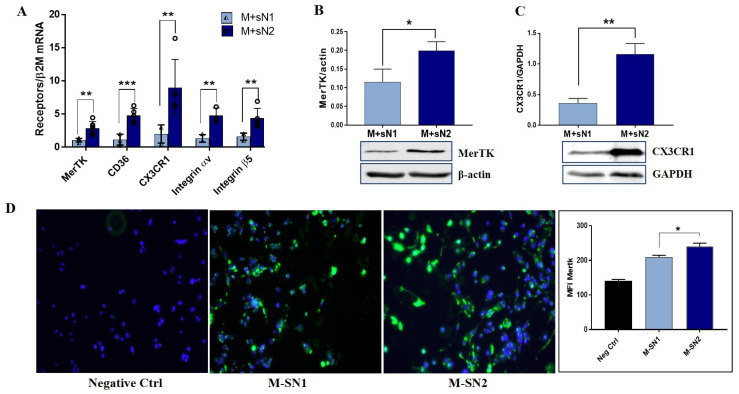
Factors released by N2 neutrophils induced upregulation of macrophage efferocytosis receptors. (**A**) Gene expressions of *MerTK*, *CD36*, *CX3CR1*, *integrin αV*, and *β5* in macrophages exposed to secretome collected from N1 (M + sN1) or N2 neutrophils (M + sN2). (**B**,**C**) Protein expressions of MerTK and CX3CR1 in the lysate of M + sN1 and M + sN2 macrophages. The data represent the mean ± SD of three different experiments; * *p* < 0.05, ** *p* < 0.01, *** *p* < 0.001 (M + sN1 versus M + sN2). (**D**) Representative images of the surface expression of MerTK on macrophages exposed to N1/N2 secretome, by immunofluorescence staining green (MerTK) and cyan (DAPI—cell nuclei), and the quantification graph.

To search for the mechanisms underlying the switch in MACs to a pro-healing phenotype, the expression of key efferocytosis signaling receptors was quantified. The results revealed that the exposure of MACs to sN2 induced a significant increase in the gene expression of all investigated receptors (i.e., *MerTK*, *CD36*, *CX3CR1*, and *integrin αVβ5*; Figure 3A) compared with the exposure to sN1. MerTK, a member of the TAM receptor tyrosine kinase family, has an essential role in macrophage efferocytosis post-MI, as it was found necessary and sufficient for efferocytosis of cardiomyocytes ex vivo [17]. Thus, by employing Western blot and immunofluorescence techniques, we found that the protein expression of MerTK also significantly increased in macrophages exposed to the N2 secretome (Figure 3B,D). Similarly, the protein expression of the CX3CR1 receptor was found to be enhanced in macrophages exposed to soluble factors released by N2 neutrophils (Figure 3C).

### 3.3. Bridging Molecules MFG-E8 and Gas6 Are Expressed by Macrophages Exposed to N2 Secretome

**Figure 4:**
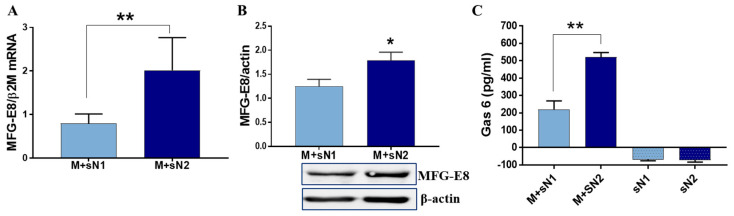
N2 secretome increased the expressions of Mfge8 and Gas6 bridging molecules. (**A**) Macrophages were exposed to conditioned media from N1/N2 neutrophils, and the *Mfge8* levels were detected by q-PCR. The relative mRNA expression is shown as the means ± SD (*n* = 3). (**B**) Western blot analysis of Mfge8 in MACs subjected to the conditions described above. The results of the densitometric analysis are shown as the means ± SD (*n* = 3). (**C**) The results of the ELISA assay for Gas6 released in the conditioned media from macrophages exposed to N1/N2 secretome are shown as the means ± SD (*n* = 4); * *p* < 0.05, ** *p* < 0.01.

The cell-surface receptors of efferocytosis engage apoptotic cells either directly, by binding to molecules on the apoptotic cells, or indirectly, by bridging molecules that mediate binding with apoptotic cells [18]. The most important bridging molecules are Gas6, protein S, and MFG-E8 [18]. Searching for the presence of these molecules, we found that when macrophages were exposed to the secretome of N1 or N2 neutrophils, the latter induced a significant modulation of surface-exposed bridging molecules. The gene and protein expressions of MFG-E8 were significantly higher in MACs exposed to sN2 compared with sN1 (Figure 4A,B). Similarly, the Gas6 level released in the conditioned media by MACs exposed to the secretome of N2 neutrophils was significantly higher than in the cells exposed to N1 secretome. None of the secretome derived from the N1/N2 neutrophil subtypes expressed detectable levels of Gas6 protein (Figure 4C), suggesting that the released Gas6 is produced by macrophages.

### 3.4. Signaling Pathway Triggered by Factors Released by N1/N2 Neutrophils

**Figure 5:**
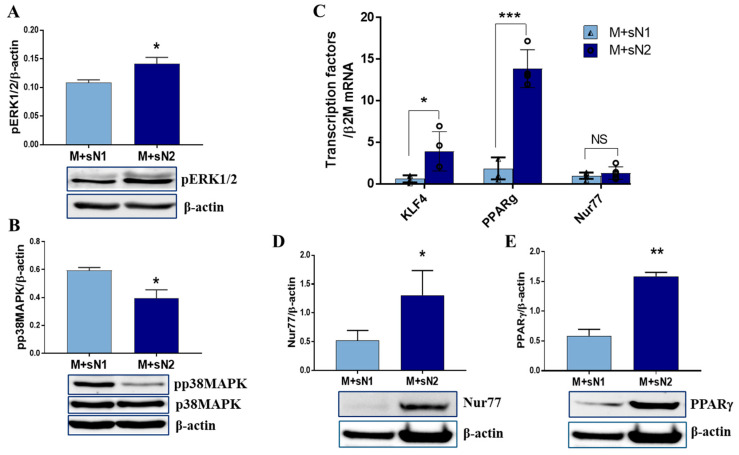
Signaling mechanism triggered by N2 mediators activated ERK1/2 and transcription factors associated with the reparatory macrophage. (**A**,**B**) Western blot analysis showed that pERK1/2 and pp38MAPM were activated differently by the N1/N2 neutrophils. The results of the densitometric analysis are shown as the means ± SD (*n* = 3). (**C**) The mRNA expressions of *KLF4*, *PPARγ*, and *Nur77* in macrophages after incubation with the secretome of N1/N2 neutrophils. The relative mRNA expression is shown as the means ± SD (*n* = 3). (**D**,**E**) Western blot analysis of the transcription factors Nur77 and PPAR-γ of macrophages exposed to N1 or N2 neutrophil secretome. The results of the densitometric analysis are shown as the means ± SD (*n* = 3). * *p* < 0.05, ** *p* < 0.01, *** *p* < 0.001.

Recently, it was found that MerTK expression and ERK1/2 activation are essential for the functional maturation of reparative macrophages after myocardial infarction [19]. The signaling mechanisms induced by neutrophil mediators in MACs were investigated by assessment of the phosphorylated forms of ERK1/2 and p38MAPK using Western blot. The results highlight that while ERK1/2 was activated in macrophages exposed to N2 secretome, pp38MAPK was downregulated (Figure 5A,B). Regarding nuclear receptors, PPARγ is a well-established master transcriptional regulator of efferocytotic macrophages [20], and KLF4 and Nur77 are associated with the reparatory phenotype of macrophages. The gene expressions of these regulatory factors were found to increase in MACs exposed to sN2 above the values obtained when cells were exposed to sN1 (Figure 5C). Moreover, the protein expression levels of Nur77 and PPARγ significantly increased in these macrophages (Figure 5D,E), which highlights their underlying reparatory phenotype.

### 3.5. Efferocytosis Capacity of Macrophages Is Increased by Factors Released by N2 Neutrophils

**Figure 6:**
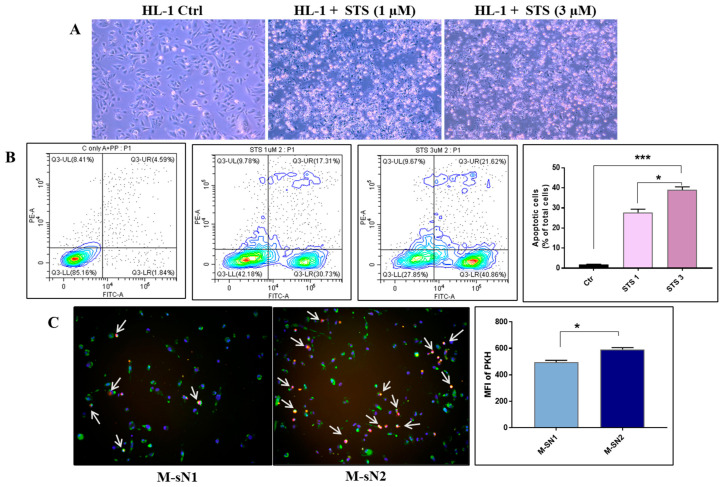
Macrophages exposed to N2 neutrophils engulf apoptotic cardiomyocytes. (**A**) Representative images of light microscopy showing the control or apoptotic HL-1 cardiomyocytes obtained by exposure to staurosporine (1 or 3 µM) for 5 h. (**B**) Flow cytometric analysis of apoptosis in human cardiomyocytes in response to staurosporine (1 and 3 µM) treatment, assessed with annexin V (FITC) and propidium iodide (PI) co-staining. Representative dot plots with the frequency of each population (pre-gated on total cells/single cells) and a quantification graph are shown (*n* = 3). * *p* < 0.05, *** *p* < 0.001. (**C**) Fluorescence microscopy showing the uptake of apoptotic cardiomyocytes (labeled with PKH-red) by human macrophages previously exposed to secretome from N1/N2 neutrophils. Macrophages were co-incubated with apoptotic cells for 2 h, and next stained for MerTK receptors (green). DAPI nuclear staining is shown in blue. White arrowheads show colocalization of apoptotic bodies with the macrophage’s MerTK receptor, indicating efferocytosis of apoptotic cardiomyocytes. The quantification graph shows the fluorescent intensity of PKH, evaluating the engulfment of apoptotic cardiomyocytes (*n* = 3, * *p* < 0.05).

To elucidate whether the efferocytosis molecules found to be increased in macrophages exposed to N2 secretome are functionally active, efferocytosis of these cells was investigated. We designed an experimental setup to obtain apoptotic cardiomyocytes by exposure to staurosporine (1–3 µM) for 5 h (Figure 6A). The flow cytometry analysis showed that the concentration of 3 µM induced ≈40% of apoptotic cells (Figure 6B). To examine the efferocytosis of apoptotic cardiomyocytes, the macrophages previously exposed to N1/N2 secretome were incubated with fluorescently labeled apoptotic cardiomyocytes, and the engulfment of apoptotic debris was quantified by fluorescence microscopy. As shown in Figure 6C, exposure to N2 secretome led to an increased number of macrophages that engulfed apoptotic debris, highlighting that the underlying efferocytosis receptors and the bridging molecules are active and functional.

## 4. Discussion

An acute inflammatory response requires a coordinated resolution program to prevent excessive inflammation, repair damage, and restore tissue homeostasis. The failure of this response contributes to the pathology of numerous chronic inflammatory diseases, including heart failure. Post-MI, high levels of necrotic and apoptotic cardiomyocytes predispose to prolonged inflammation. Impaired clearance of dying cardiomyocytes promotes further collateral cell death and permanent loss of their contractile function. Therefore, the clearance of dying cardiomyocytes or cell debris (i.e., efferocytosis) is a fundamental function of MACs.

Macrophages respond dynamically to changes in the environment by exhibiting different phenotypes. In the case of MI, 1–3 days after a mouse infarct, infiltrating MACs exhibited a pro-inflammatory phenotype, and at days 5–7 post-MI, the cells adopted a reparative M2-like phenotype displaying increased efferocytosis capacity and were involved in inflammation resolution [21]. As the macrophages are exposed in the first days to inflammatory neutrophils and, starting on day 5, to the anti-inflammatory neutrophil subtype [11], here, we expand on the observations that macrophages are sensitive to environmental factors and question the effect of factors released by the two neutrophil subtypes on MACs.

**Figure 7:**
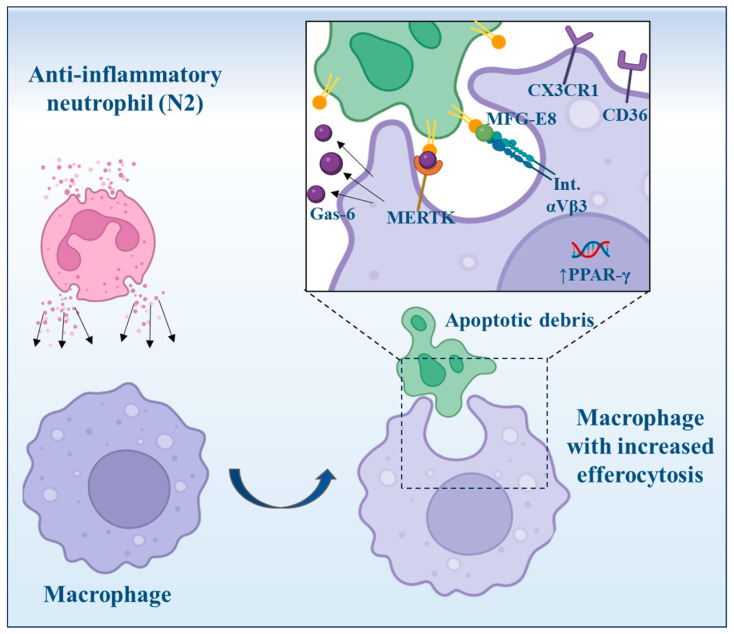
Factors released by N2-anti-inflammatory neutrophils induced a reparatory M2-like phenotype in macrophages (image created with BioRender).

The novel data reported here demonstrate that factors released by N2-anti-inflammatory neutrophils induce a reparatory M2-like phenotype in macrophages derived from THP1 monocytes, which displayed increased expressions of the efferocytosis receptors MerTK, *CD36*, CX3CR1, and *integrin αV/β5* and the bridging molecules Mfge8 and Gas6, as well as the ensuing enhanced functional efferocytosis activity (Figure 7).

Macrophages have key roles in inflammation and its resolution. Post-MI, infiltrated neutrophils degranulate, and along with DAMPs released by necrotic cardiomyocytes, amplify the inflammatory response by further recruiting immune cells, including monocytes that differentiate into MACs. Changing cues in the inflammatory milieu alter macrophage programming and modulate their various endocytic functions. An important change signal in the infarcted area are the neutrophils, which temporarily change their phenotype [11].

To evaluate the impact of factors released by polarized neutrophils on the macrophage phenotype, our initial focus was on the pro-inflammatory and anti-inflammatory markers. The increased expression of pro-inflammatory markers, such as TNF-α, IL-1β, and S100A8/A9, in the macrophages exposed to the secretome from N1 neutrophils indicates that factors released by these cells promote an M1-like inflammatory response in macrophages. In contrast, the increased expression of the anti-inflammatory markers CD206, TGF-β, and IL-10 by N2 in the MACs exposed to the N2 secretome indicates that factors released by N2 cells promote an M2-anti-inflammatory response. These results are in good agreement and extend previous reports showing that neutrophils shape monocyte differentiation and macrophage polarization. Thus, it was reported that azurocidin and lactoferrin produced by neutrophils during infection induce the polarization of MACs to a proinflammatory M1 phenotype [22], characterized by TNFα and interferon (INF)γ release and enhanced phagocytosis [23]. Moreover, S100A9—an alarmin mainly produced by activated neutrophils—induces the secretion of proinflammatory cytokines and matrix metalloproteinases (MMPs) by synovial MACs in osteoarthritis [24]. These reports demonstrate that the inflammatory N1 neutrophils (recruited in the acute phase of inflammation) polarize macrophages toward an M1 inflammatory phenotype. Our new data extend these results, showing that factors released by the anti-inflammatory subset of neutrophils (found in the reparatory phase post-MI) produce increased levels of IL-10 and TGF-β in MACs derived from THP-1 monocytes. These anti-inflammatory mediators are known to dampen the pro-inflammatory responses, a characteristic of MACs that promotes tissue repair [25].

One of the main functions of the MACs is efferocytosis, a process that is regulated by several recognition and uptake receptors. Different efferocytosis receptors have been identified to date and include a structurally heterologous group of surface proteins that bind either directly to phosphatidylserine (MerTK, CD36, CD300, and integrin αV/β5) or indirectly via recognition of soluble opsonins (MFG-E8, CCN1, Gas6, and protein S), which bind to phosphatidylserine (PS) [26]. Among them, the MerTK expressed by MACs is considered one of the main receptors of efferocytosis [27]. By interacting with Gas6 or protein S, bridging molecules that bind externalized PS on apoptotic cells, MerTK plays an important role in inflammation resolution and efferocytosis [17]. Binding to MerTK triggers the internalization of apoptotic cells via cytoskeletal signaling and activates an anti-inflammatory response by suppressing NF-κB [28]. Mice lacking macrophage MerTK have an exaggerated response to ischemia–reperfusion injury of the left anterior descending artery, characterized by the accumulation of apoptotic cells and larger infarct size [29]. Importantly, in an MI murine model induced by ligation of the left anterior descending coronary artery, the depletion of neutrophils led to a deterioration in cardiac function, increased fibrosis, and a gradual elevation in heart-failure-related biomarkers [10]. These effects were accompanied by a decline in the cardiac expression of MerTK on macrophages [17].

To identify the underlying mechanism of the occurrence of the pro-healing phenotype of M2 macrophages following exposure to sN2, we set up experiments to assess the presence of the specific molecules involved in the efferocytosis process. Exploring the receptors MerTK, CD36, CX3CR1, and integrin αV/β5, we found that all these receptors exhibited a significantly increased expression in MACs exposed to sN2 compared with MACs exposed to sN1, with the data suggesting that factors released by anti-inflammatory neutrophils upregulate efferocytosis receptors. As mentioned, some of the receptors bind to PS indirectly with the assistance of bridging molecules, such as MFG-E8, Gas6, and protein S. These bridging molecules have been extensively documented to play a pivotal role in ensuring the efficient clearance of cells [30]. Therefore, upon binding to PS on apoptotic cells, MFG-E8 is recognized by the αvβ3 integrin receptor on phagocytes, leading to Rac activation and the internalization of apoptotic bodies, limiting tissue inflammation [31]. Moreover, MFG-E8 mediates the interactions between the macrophage MerTK and apoptotic cells, improving cardiac repair after MI [32]. Our experiments revealed a significant elevation of both MFG-E8 and Gas6 in M + sN2 as compared to the M + sN1, with the data suggesting that, in addition to the increased efferocytosis receptors, N2 neutrophils also play a role in the upregulation of bridging molecules in macrophages derived from THP1 monocytes. Importantly, the receptors and bridging molecules involved in the efferocytosis process, as well as the marker of the M2 macrophage, IL-10, were similarly increased by N2 secretome in other human macrophages derived from U937 monocytes (Supplementary Materials Figure S2).

For a complete view of the neutrophils’ impact on macrophages, we investigated the cellular signaling pathways induced by mediators released from the N1/N2 neutrophils and found activation of ERK1/2 by sN2 and of p38MAPK by sN1, results that were previously associated with M2 MACs. Thus, IL-10 and MCSF were found to activate STAT3 and ERK1/2 in cardiac macrophages, which, in turn, upregulate the expression of galectin-3 and MerTK, leading to the functional maturation of reparative macrophages [19]. Moreover, the activation of MerTK in MACs led to ERK1/2 activation and reduced activity of p38MAPK, resulting in enhanced biosynthesis of pro-resolving mediators [33]. An analysis of the nuclear receptors PPARγ, KLF4, and Nur77 revealed their increased levels in macrophages exposed to sN2. Recent evidence strongly supports the role of these transcription factors in promoting a pro-healing phenotype in macrophages. The nuclear receptor Nur77 induces an anti-inflammatory metabolic state in macrophages that protects against chronic inflammatory diseases, such as atherosclerosis [34]. KLF4 is involved in skewing the macrophages to the M2 phenotype, and nuclear receptor PPARγ controls the expression of M2-associated genes [35,36]. In addition, our data demonstrate that PPARγ is involved in the regulation of efferocytosis-associated genes, as the MerTK and CX3CR1 protein expressions were significantly reduced in the presence of the PPARγ antagonist GW9662 (Supplementary Materials Figure S3). These results are in good agreement and extend previous data showing that activation of PPARγ leads to the regulation of efferocytosis and production of pro-resolving cytokines [37]. As the factors released by anti-inflammatory neutrophils increased both efferocytosis receptors and PPARγ, we finally investigated the impact of these factors on the efferocytosis process, per se. The results corroborate well, showing that the macrophages pre-exposed to sN2 exhibited a higher clearance of apoptotic cardiomyocytes compared to those exposed to sN1. Although previous work has shown that pro-inflammatory neutrophils (present in the acute phase of inflammation) polarize macrophages toward an M1 inflammatory phenotype with enhanced microbicidal activity [22], our data demonstrate that factors released by the anti-inflammatory subset of neutrophils (found in the reparatory phase post-MI) induce a pro-healing macrophage phenotype with increased efferocytosis activity. Regarding the specific factors present in the N2 neutrophil secretome that may underlie these effects, IL-10 is one possible candidate, as previously it was found to induce macrophage efferocytosis [38,39], and we found that IL-10 increased in the human N2 neutrophils (Supplementary Materials Figure S1), but further experiments are needed to clarify this matter.

## 5. Conclusions

In conclusion, these data demonstrate the role of factors released by anti-inflammatory N2 neutrophils in inducing a reparatory phenotype in macrophages by inducing the anti-inflammatory molecules CD206, TGF-β, and IL-10; nuclear factors associated with the reparatory macrophages, PPARγ, Nur77, and KLF4; as well as molecules associated with the efferocytosis process, including MerTK, Mfage8, and Gas6 molecules. Together, our comprehensive data uncover a remarkable and previously unrecognized function of an anti-inflammatory N2 neutrophil subtype: to significantly amplify the macrophages’ capacity to carry out efferocytosis, an essential mechanism for resolving inflammation. Given the crucial role of pro-healing M2 macrophages in cardiac healing during the reparative phase, targeting N2 neutrophils with this specific phenotype could hold promise as a post-MI therapeutic strategy.

## Data Availability

The data presented in this study are available on request from the corresponding author.

## Supplementary Materials

The following supporting information can be downloaded at: https://www.mdpi.com/article/10.3390/cells13030208/s1, Table S1. The sequences of oligonucleotide primers used for evaluation of gene expression. Figure S1. Inflammatory/anti-inflammatory markers in human neutrophils polarized in N1 or in N2 neutrophils. A–E. Gene expression of inflammatory markers TNFα, IL-12, IL-1β, CCL3, and CCL4 in human neutrophils polarized for 2 h with LPS + IFNγ (N1) or IL-4 (N2), and in control neutrophils (C). **F**, **G**. Gene expression of anti-inflammatory markers IL-10 and CD206, in N1, N2 and C neutrophils. n = 5, * *p* < 0.05, ** *p* < 0.01, *** *p* < 0.001, (N1 vs. N2). Figure S2. Effect of N1/N2 neutrophil secretome on human macrophages derived from U937 monocyte line. A–C. Gene expression of receptors involved in efferocytosis process MerTK, integrin β5, and CX3CR1; D. Gene expression of MFG-E8 bridge molecule; E. Gene expression of PPARγ transcription factor, and F. Gene expression of IL-10 anti-inflammatory molecule in macrophages derived from U937 exposed to N1/N2 conditioned media. n = 2 (2 experiments with 2 wells/experiment), * *p* < 0.05, ** *p* < 0.01, (N1 vs. N2). Figure S3. PPARγ inhibitor GW9662 reduces the expression of MerTK and CX3CR1 efferocytosis receptors. (A, C) Protein expression of MerTK, MFG-E8, and CX3CR1 in lysate of macrophages exposed to sN2 in the absence (MN2) or presence of 10 µM GW9662 inhibitor (MN2+GW). The data represent mean ± SD from three different experiments, * *p* < 0.05.
