# Supplementary material for: Anti-Inflammatory Neutrophils Reprogram Macrophages toward a Pro-Healing Phenotype with Increased Efferocytosis Capacity
Source: Cells. 2024 Jan 23;13(3):208. doi: 10.3390/cells13030208 (PMC10854927; doi:10.3390/cells13030208)
Supplement: Supplementary file 1 [file cells-13-00208-s001.zip › cells-2763122-supplementary.pdf]

Supplementary material

Supplementary table S1:

Table S1. The sequences of oligonucleotide primers used for evaluation of gene expression.

Gene (Human)	Sequences of Oligonucleotide Primers
TNFα	GGTGCCTATGTCTCAGCCTCT<br>CATCGGCTGGCACCACTAGTT
IL1β	AGAGTGGAGCCTGGTCTTACA<br>CCTTTGCTGACAATAAGCACTGG
S100A8	ATGCCGTCTACAGGGATGAC<br>ACTGAGGACACTCGGTCTCTA
S100A9	TCCTCGGCTTTGACAGAGTG<br>GCCCCAGCTTCACAGAGTAT
CD206	GGGTTGCTATCACTCTCTATGC<br>TTTCTTGTCTGTTGCCGTAGTT
IL10	GCTCTTACTGACTGGCATGAG<br>CGCAGCTCTAGGAGCATGTG
TGF-β	CAAGCAGAGTACACACAGCAT<br>TGCTCCACTTTTAACTTGAGCC
MerTK	CTCTGGCGTAGAGCTATCACT<br>AGGCTGGGTTGGTGAAAACA
CD36	AAGCCAGGTATTGCAGTTCTTT<br>GCATTTGCTGATGTCCAGCACA
CX3CR1	CACACCGTCATCAGCATTGA<br>GGTAGTCACCAAGGCATTCATT
Integrin αv	GCTGTCGGAGATTTCAATGGT<br>TCTGCTCGCCAGTAAAATTGT
Integrin β5	GGAAGTTCGGAAACAGAGGGT<br>CTTTCGCCAGCCAATCTTCTC
MFG-E8	CCTGCCACAACGGTGGTTTAT<br>CACATTTCGTCTCACAGTGGTT
KLF	CAGCTTCACCTATCCGATCCG<br>GACTCCCTGCCATAGAGGAGG
PPARγ	ACCAAAGTGCAATCAAAGTGGA<br>ATGAGGGAGTTGGAAGGCTCT
Nur77	GGCTCGGGGATACTGGATACA<br>CTGGCATGAAGCGTTGTCC

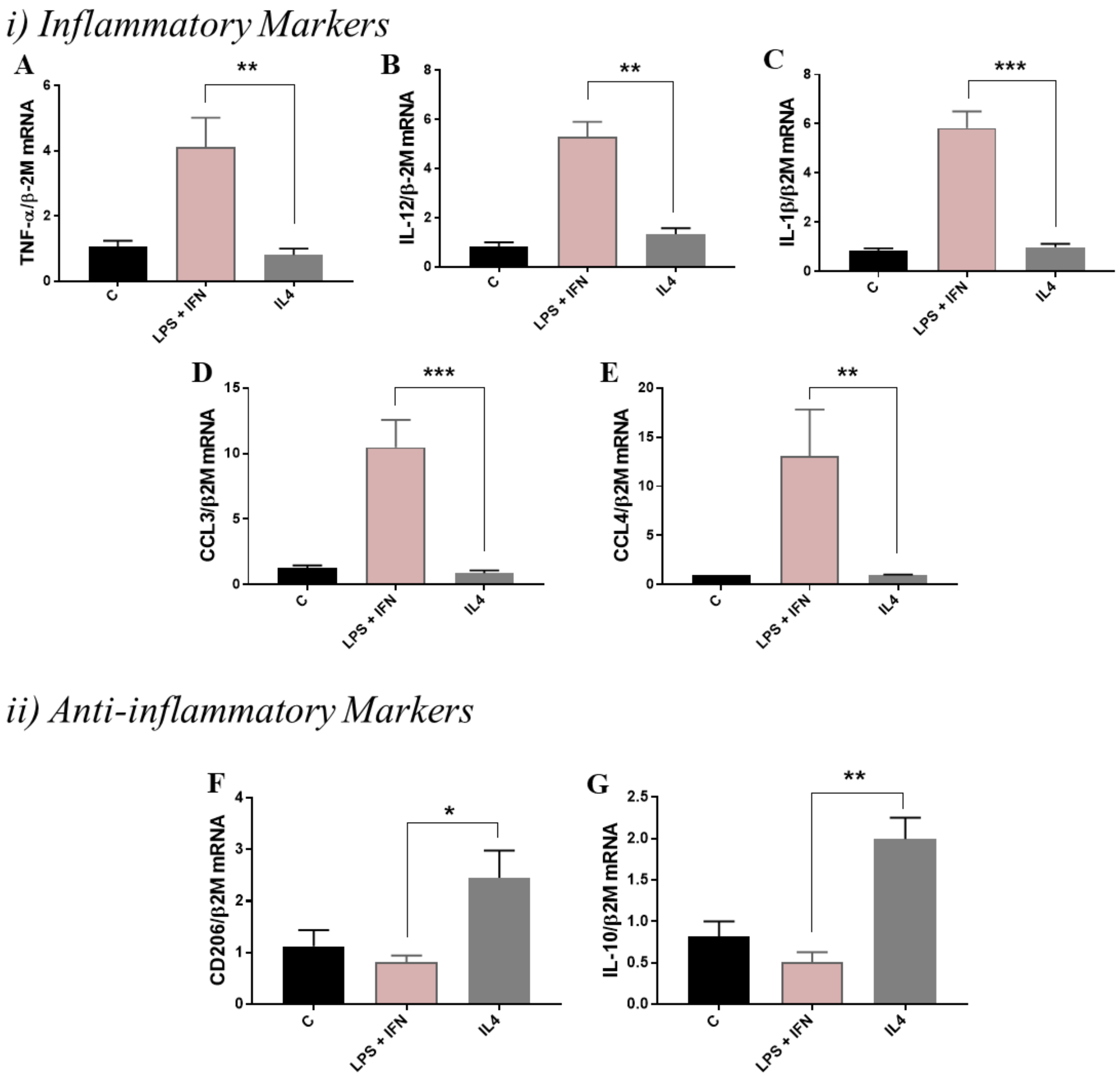

Supplementary Figure S1. ***Inflammatory/anti-inflammatory markers in human neutrophils polarized in N1 or in N2 neutrophils*****. A – E.** Gene expression of inflammatory markers TNFα, IL-12, IL-1β, CCL3, and CCL4 in human neutrophils polarized for 2h with LPS+IFNγ (N1) or IL-4 (N2), and in control neutrophils (C). **F, G.** Gene expression of anti-inflammatory markers IL-10 and CD206, in N1, N2 and C neutrophils. n=5, *$p<0.05$, **$p<0.01$, ***$p<0.001$, (N1 vs. N2).

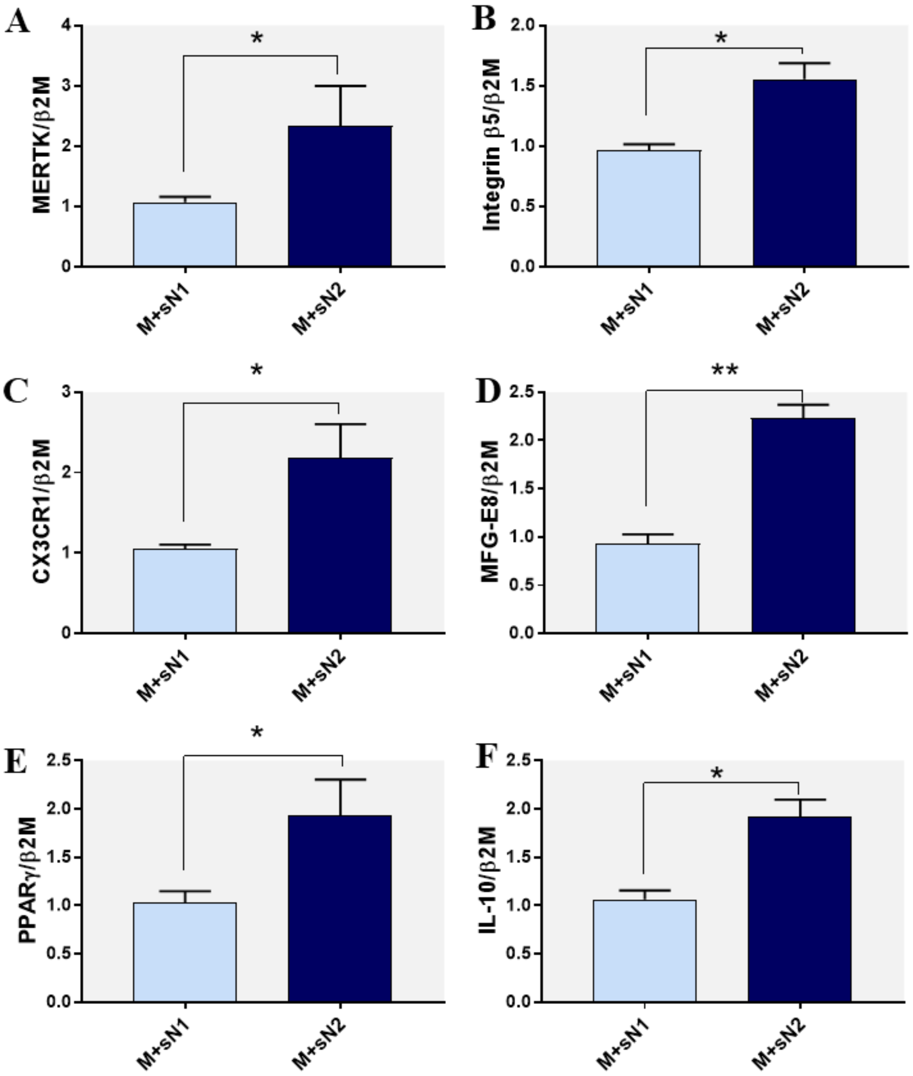

Supplementary Figure S2. ***Effect of N1/N2 neutrophil secretome on human macrophages derived from U937 monocyte line***. **A – C.** Gene expression of receptors involved in efferocytosis process MerTK, integrin β5, and CX3CR1; **D.** Gene expression of MFG-E8 bridge molecule; **E.** Gene expression of PPARγ transcription factor, and **F.** Gene expression of IL-10 anti-inflammatory molecule in macrophages derived from U937 exposed to N1/N2 conditioned media. n=2 (2 experiments with 2 wells/experiment), *p<0.05, **p<0.01, (N1 vs. N2).

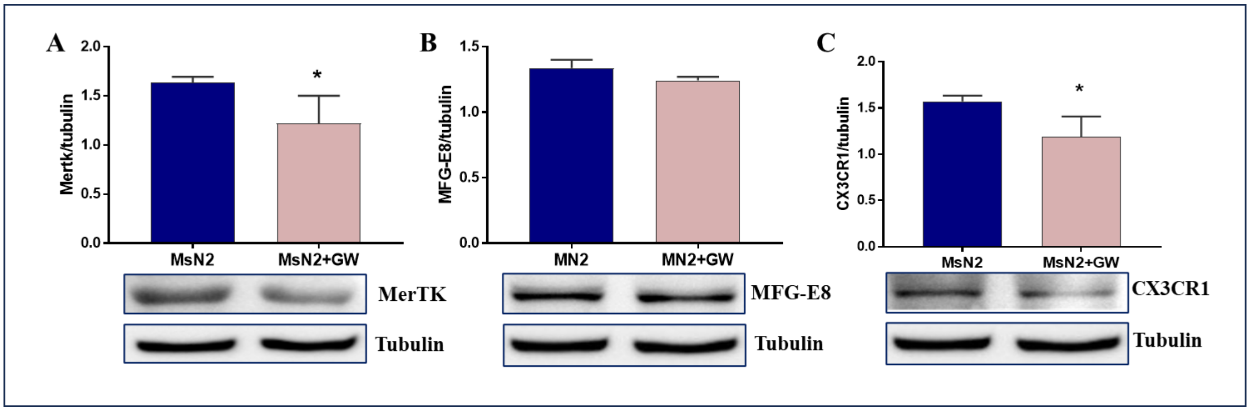

Supplementary Figure S3. ***PPARγ inhibitor GW9662 reduces the expression of MerTK and CX3CR1 efferocytosis receptors. (A, C)*** *Protein expression of MerTK, MFG-E8, and CX3CR1 in lysate of macrophages exposed to sN2 in the absence (MN2) or presence of 10µM GW9662 inhibitor (MN2+GW). The data represent mean ± SD from three different experiments, *p<0.05.*
